# GPER-Deficient Rats Exhibit Lower Serum Corticosterone Level and Increased Anxiety-Like Behavior

**DOI:** 10.1155/2020/8866187

**Published:** 2020-08-28

**Authors:** Yi Zheng, Meimei Wu, Ting Gao, Li Meng, Xiaowei Ding, Youqiang Meng, Yingfu Jiao, Ping Luo, Zhenquan He, Tao Sun, Guohua Zhang, Xueyin Shi, Weifang Rong

**Affiliations:** ^1^Department of Anesthesiology, Xinhua Hospital, Shanghai Jiaotong University School of Medicine, Shanghai, China; ^2^Department of Anatomy and Physiology, Shanghai Jiaotong University School of Medicine, Shanghai, China; ^3^Department of Anesthesiology, Renji Hospital, Shanghai Jiaotong University School of Medicine, Shanghai, China; ^4^Key Laboratory of Cerebrocranial Diseases, Ningxia Medical University, Yinchuan 750004, China

## Abstract

Ample evidence suggests that estrogens have strong influences on the occurrence of stress-related mood disorders, but the underlying mechanisms remain poorly understood. Through multiple approaches, we demonstrate that the G protein-coupled estrogen receptor (GPER) is widely distributed along the HPA axis and in brain structures critically involved in mood control. Genetic ablation of GPER in the rat resulted in significantly lower basal serum corticosterone level but enhanced ACTH release in response to acute restraint stress, especially in the female. GPER^−/−^ rats of either sex displayed increased anxiety-like behaviors and deficits in learning and memory. Additionally, GPER deficiency led to aggravation of anxiety-like behaviors following single-prolonged stress (SPS). SPS caused significant decreases in serum corticosterone in WT but not in GPER-deficient rats. The results highlight an important role of GPER at multiple sites in regulation of the HPA axis and mood.

## 1. Introduction

Women are at least twice as likely to develop mood disorders such as anxiety, depression, and posttraumatic stress syndromes [1–3]. Such gender-related differences in the occurrence of mood disorders manifest after puberty, implicating an important role of the female hormone estrogens in modulation of anxiety [4, 5]. Indeed, numerous clinical and preclinical observations have indicated a strong interaction between mood and estrogen levels in females. Paradoxically, however, in most human studies, high and constant estrogen levels have been described as anxiolytic and “emotionally positive,” whereas low or fluctuating estrogen levels have been shown to correlate with increased anxiety [6–8]. Similarly, in rodents, low estrogen levels have been associated with increased anxiety and exogenous estrogen administration has been shown to alleviate anxiety [9–13]. These findings suggest that estrogens may play important but complicated roles in the regulation of mood. Understanding the mechanisms of estrogenic actions in mood regulation may be important for the prevention and effective management of mood disorders, particularly in the female population.

Mood disorders are closely related to abnormalities in the stress responses, which are also sex-biased and are modulated by estrogens [14, 15]. It has been well documented that stressful events trigger negative emotions with greater intensity in women than in men [16, 17], which may be attributable to differences in the corticolimbic circuitry comprising the amygdala, prefrontal cortex and the hippocampal formation [18, 19]. The autonomic and neuroendocrine responses to stress are also sex-biased, such that females generally have higher basal glucocorticoid levels and greater increases in glucocorticoid release in response to stress than males, whereas basal and stress-induced adrenaline release is lower in females than in males [2, 20].

Until now, at least three types of estrogen receptors have been identified: the nuclear receptors ER*α* and ER*β*, which mediate slow genomic effects, and the G protein-coupled estrogen receptor (GPER or GPER-1, formerly known as GPER30), which mediates rapid nongenomic effects [21]. The distribution of ER*α* and ER*β* within the CNS and their roles in sex-biased stress responses or mood disorders have been studied extensively, with recent evidence suggesting sex-specific involvement of ER*α* and ER*β* in behavioral responses to stress [22]. ER*β* likely plays an anxiolytic role since E2 had an antianxiety effect in wild-type but not in ER*β* knockout mice [23]. It has also been reported that serum concentration of corticosterone was increased in ER*β* knockout mice [24], whilst ER*α* knockout mice had similar serum corticosterone level as WT mice at least in the male [25].

On the other hand, the involvement of GPER in sex-biased stress responses has not been as vigorously investigated, although immunohistochemical mapping indicated widespread distribution of GPER in the CNS. High-level expression of GPER or GPER mRNA has been reported in the cortical, the hippocampus, the amygdala, and the hypothalamus, but the results were not always consistent [26–28]. Moreover, although several studies have implicated GPER in the modulation of anxiety, there have been conflicting reports as to whether this receptor is anxiogenic or anxiolytic. For example, Hart et al. reported that systematic application of the GPER agonist G-1 led to a decrease in anxiety-like behavior in gonadectomized male mice without significant effect in ovariectomized female mice [29]. In contrast, Kastenberger et al. reported an increase in anxiety-like behavior following systematic G-1 treatment in ovariectomized mice and intact male mice in the elevated plus maze and open field test [30]. A subsequent follow-up study by the same group revealed a phenotype of reduced anxiety-like behavior in male but not female GPER knockout mice [31]. Therefore, further studies are needed in order to determine the distribution of GPER in the CNS and its role in the regulation of stress response and anxiety.

In the current investigation, complementary measures were taken to systematically analyze the distribution of GPER in the corticolimbic circuit and the HPA axis. The possible role of GPER in the regulation of anxiety-like behaviors and the HPA axis was explored using GPER-deficient (GPER^−/−^) rats. Our results indicate that GPER is anatomically positioned to influence the cognitive, autonomic, and neuroendocrine responses to stress and plays prominent roles in the regulation of anxiety.

## 2. Material and Methods

### 2.1. Animals

Gper-Cre transgenic mice were generated in Shanghai Model Organisms Inc. (Shanghai) with a knock-in of the 2A-Cre gene fragment into the GPER gene stop codon based on CRISPR/Cas9 system. GPER reporter (GPER Cre/tdTomato) mice were obtained by crossing the Gper-Cre mice with Ai14(RCL-tdT)-D mice. GPER-deficient Sprague Dawley (SD) rats (GPER^−/−^ rats, with a 139 bp deletion of GPER gene, Gene ID 171104) were generated through the CRISPR/Cas9 gene-editing approach in BIORAY BIOTECHNOLOGY (Shanghai, China), which has been described previously [32]. Age-matched WT SD rats were provided by Shanghai Jiaotong University School of Medicine. The animals were housed (5 per cage) in an air-conditioned room (23°C with 60% humidity) with a 12 h light-dark cycle (lights on 7 a.m. to 7 p.m.) and free access to food and water. Cages were changed weekly and no more than 48 h before any behavioral test. All the experimental procedures were in compliance with the Guiding Principles in the Care and Use of Animals and the Animal Management Rule of the Ministry of Public Health, People's Republic of China (documentation 545, 2001), and had been approved by the Institutional Ethic Committee for Experimental Use of Animals of Shanghai Jiaotong University School of Medicine (document #SYXK-2013-0050). Every effort was taken to minimize the number of animals used.

### 2.2. Ovariectomy

Bilateral ovariectomy (OVX) was performed on a group of female rats at the age of 8 weeks under anesthesia (ketamine 25 mg/kg, ip for induction, and 2% sevoflurane for maintenance of anesthesia) and aseptic condition. Their back was cleaned and shaved, and a 1 cm incision was made in the skin. Incisions were made bilaterally in the muscle above the ovaries, the ovaries were drawn out and clamped at the uterus, and the ovary was incised above the clamp. The uterus was then put back into the abdominal cavity, and the incision in the skin was closed with 1 or 2 MikRon wound clips (MikRon Precision Inc., Gardena, CA). A single dose of 2 mg/kg meloxicam was administered for postsurgical analgesia. Rats were single housed following surgery and were allowed 2 weeks of recovery before behavior tests.

### 2.3. Single-Prolonged Stress (SPS) Model

Eight-week-old GPER^−/−^ and WT rats of either sex were randomly assigned to the SPS or control groups. SPS rats were exposed to three consecutive stressors: two hours of restraint, followed by 20 min of forced swimming and 5 min of general anesthesia with sevoflurane. Rats were then returned to their home cages for a 14-day quiescent period. The control groups were left undisturbed in their home cages for the duration of the experiment.

### 2.4. Immunofluorescence and Nissl Staining

Adult (16 weeks of age) WT or GPER^−/−^ rats or mice were euthanized by an overdose of sodium pentobarbital and were immediately perfused with phosphate-buffered saline (PBS) followed by 4% paraformaldehyde (PFA). The brain was dissected out and postfixed in 4% PFA. After fixation, the tissue was dehydrated in 30% sucrose solution for 24 h and embedded in OCT on the dry ice. For immunofluorescence, the brain was cut into 15 *μ*m thick coronal sections. Sections were blocked with 5% normal donkey serum (NDS, Interchim) in PBS for 1 h and were then incubated with the primary antibodies (anti-GPER, 1 : 300, #A4272, Lifespan, Seattle, WA; anti-S100*β*, 1 : 300, #ab52642, Abcam, Cambridge, MA) diluted in PBS containing 1% NDS. Sections were washed 4 times with PBS, then incubated with the secondary antibodies (Alexa Fluor-conjugated goat anti-rabbit IgG, 1 : 1000, #A11304, Invitrogen, Carlsbad, CA) for 2 h at room temperature. Sections were examined with a LEICA DM 2500 microscope equipped for epifluorescence. Nissl staining was conducted on 30 *μ*m brain slices following the protocol described by Mantamadiotis et al. [33].

### 2.5. GPER RNAscope In Situ Hybridization

#### 2.5.1. Tissue Preparation

WT or GPER Cre/tdTomato mice were euthanized by an overdose of sodium pentobarbital and were perfused transcardially with normal saline and 4% PFA (freshly prepared with PBS). Tissues (brain, pituitary, adrenal gland, or sympathetic ganglia) were collected and postfixed in 4% PFA at 4°C for 24 h, thereafter transferred to 30% sucrose solution until the tissues sank to the bottom of the container. The tissues were embedded with OCT and stored at -80°C. For GPER RNAscope in situ hybridization, the tissue mass was equilibrated at -20°C for at least 1 h and was subsequently cut into 15 *μ*m thick sections using a cryostat. Tissue sections were mounted onto slides, equilibrated at -20°C for 20 min, and then dried at 60°C for 30 min. The sections were then fixed in freshly prepared 4% PFA for 15 min, washed in PBS for 5 min to remove OCT, treated with hydrogen peroxide for 10 min, and subsequently washed with distilled water. The slides were submerged into the target repair reagent (preheated to 98-102°C) for 5 min, washed with distilled water 3 times, treated with 100% ethanol for 3 min, and then dried at room temperature. The tissue sections were covered with Drop protease III reagent, and the slides were left in HybEZ™ hybrid oven (40°C, preheated for 30 min) for 20 min. The slides were washed with distilled water 3 times.

#### 2.5.2. RNAscope® In Situ Hybridization

RNAscope® multichannel fluorescent second generation kit (cat. 323100), customized double Z oligonucleotide probes including Probe-Mm_gpr30 (cat.318191), Duplex Positive Control Probe (cat. 321651), and 2-plex Negative Control Probe (cat. 320751) were all purchased from Advanced Cell Diagnostics (ACD, USA). Opal fluorescent dye (Opal520) was from PerkinElmer. We followed the instructions accompanying the kit to carry out target probe hybridization (only one probe is used for each tissue section), hybridization signal amplification, and probe signal marking using Opal520. Sections were examined under a LEICA DM 2500 microscope.

### 2.6. Determination of Serum Levels of Stress Hormones

Rats were killed by decapitation. Blood samples were collected in blank EP tubes (for serum separation) or in EP tubes containing aprotinin 0.6 TIU/ml and saturated EDTA-Na2 (for plasma separation). For serum separation, the blood samples were kept at 4°C overnight and then centrifuged (1500 rpm) at 4°C for 20 min. For plasma separation, the blood samples were immediately centrifuged (1500 rpm) at 4°C for 20 min. Serum and plasma were collected into new EP tubes and stored at -80°C until assay.

Serum levels of endogenous corticosterone, 17*β*-estradiol, and adrenaline were determined by liquid chromatography-mass spectrometry (LC-MS). Briefly, 100 *μ*l serum was added to 400 *μ*l precooled acetonitrile and vortexed for 30 s. The mixture was centrifuged for 10 min at 4°C, 150000 g, and the supernatant was vacuum dried and redissolved in 150 *μ*l of 50% acetonitrile solution. After being vortexed for 60 s, the sample was centrifuged for 10 min (150000 g, 4°C). 100 *μ*l of the supernatant was collected, filtered through a 0.22 *μ*m filter, and then entered into liquid chromatography tandem mass spectrometry for quantitative analysis. Concentration of corticosterone, estradiol, and adrenaline in each sample was extrapolated from their respective standard curves (10, 20, 50, 100, 200, 500, and 1000 ng/ml for corticosterone and adrenaline; 0.2, 1, 2, 5, 10, 20, 50, and 100 ng/ml for estradiol).

Plasma levels of peptide hormones were determined by ELISA. The detection sensitivity of the ELISA kits (all from Phoenix Pharmaceuticals, Inc., Burlingame, CA, USA) was 0.33 ng/ml for CRH (cat. EK-022-33), 0.08 ng/ml for ACTH (cat. EK-001-21), 0.04 ng/ml for AVP (cat. EK-065-07), and 0.17 ng/ml for *β*-endorphin (cat. EK-022-33).

### 2.7. Elevated Plus Maze (EPM) Test

The EPM consisted of 4 arms, forming the shape of a plus, elevated 70 cm above the floor. Two opposing arms were closed by black walls; the other two arms were open. All four arms were connected by a neutral field. The dimensions were 30 × 5 cm for the arms and 5 × 5 cm for the neutral field, and the framing of the closed arm had a height of 15 cm. Illumination in the neutral field was set to 180 lx. Each rat was placed gently on the neutral field facing an open arm and allowed to explore the maze for 5 min. The time spent and the number of entries into the open arm were taken as measures of trait anxiety levels.

### 2.8. Open Field Test

The test apparatus was a 70 × 70 cm synthetic box. The arena was divided into 3 areas. The border area was 15 cm from the wall, the center (25 × 25 cm) covered 13% of the total area, and the area in-between was the intermediate zone. Illumination was set to 150 lx in the center of the open field. When tested, each rat was placed in the middle of the open field and recorded for 5 min. The time the rat spent, the distance traveled, and the number of visits to the center of the open field were taken as measures of anxiety levels.

### 2.9. Morris Water Maze

A white circular tank (150 cm in diameter, 80 cm in height) was filled with water (24°C, 60 cm in height) and was surrounded by a variety of extra maze cues. The tank was divided into four quadrants, and four start positions were located at the intersections of the quadrants. A platform (10 cm in diameter, 2 cm beneath the water) was placed singly in the center of the quadrants. Data were recorded using an automated tracking system. The protocols include the following. (1) Adaptive training: one day before the experiment, animals were forced to swim in the water without platform twice (90 s each time). (2) Positioning navigation: the experiment lasted five days. On each testing day, the rat was put into the water atone quadrant facing the wall of the pool. When the rat found the platform, it was allowed to stand on the platform for 30 s. The rat was then taken off the platform and allowed to rest for 60 s. The experiment was repeated three times by placing the rat into the water at another random quadrant. If the platform could not be found within 90 s, the rat was guided to the platform and rested for 30 s, and the latency was recorded as the highest score of 90 s. The time of rats finding the platform (escape latency) was recorded. (3) Space exploration: on the sixth day, the platform was withdrawn and the rats were placed into the pool at random in a quadrant; the time of rats swimming in the quadrant of the platform within 90 s was recorded.

### 2.10. IntelliCage

IntelliCage was used to study individual animal's behaviors related to anxiety, learning, and memory in a social environment. Before the test, each rat (WT or GPER^−/−^) was implanted with a unique microchip, allowing individual animal's behavior to be registered. The cage (140 × 140 × 45 cm) is equipped with four operant conditioning chambers located in each corner. Each conditioning chamber contains two drinking bottles accessible by a small opening containing a transponder reader antenna that registers the microchip of the entering rat. Access to each water bottle is controlled by a nosepoke hole containing infrared beam-break sensors, which can be programmed to open or remain closed upon visit or nosepoke. There is also a high-pressure jet at the opening of each corner, which can punitively spray animals when needed.

#### 2.10.1. Learning and Memory-Related Behavior Test

**Figure 1:**
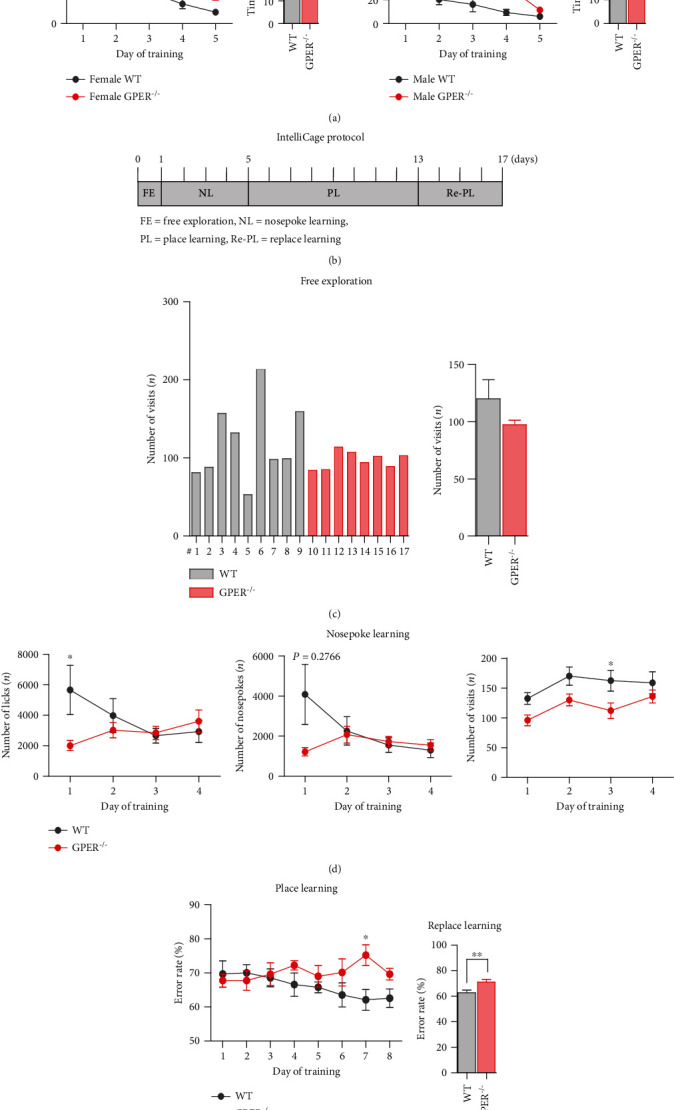
GPER-deficient rats exhibit deficits in learning and memory. (a) Comparison of the latency to find the platform and the percentage of time in the target quarter in Morris water maze test between WT and GPER^−/−^ rats (*n* = 7 for each group). Note that in the positioning navigation period, GPER^−/−^ rats spent more time to find the platform than WT rats. (b) Protocol of the IntelliCage test to evaluate learning and memory of female WT (*n* = 9) and GPER^−/−^ (*n* = 8) rats. (c) Comparison of the number of corner visits in IntelliCage free exploration (FE) period between female WT and GPER^−/−^ rats. The left panel shows the number of corner visits of individual rats, and the right panel shows the averaged number of corner visits of WT and GPER^−/−^ rats. (d) Comparison of the number of licks, nosepokes, and visits in the nosepoke learning (NL) period between female WT and GPER^−/−^ rats. Note that GPER^−/−^ rats showed fewer number of licks, nosepokes, and visits than WT rats, indicating decreased basic skill learning ability. (e) Comparison of the error rate of corner visits in the place learning (PL) period between WT and GPER^−/−^ rats, with the GPER^−/−^ group showing higher error rate of corner visits. (f) Comparison of the error rate of corner visits in the replace learning (Re-PL) period between WT and GPER^−/−^ rats, with the GPER^−/−^ rats showing higher error rate than WT rats. ∗*P* < 0.05, ∗∗*P* < 0.01, and ∗∗∗*P* < 0.001, unpaired *t*-test.

Ten-week-old female GPER^−/−^ (*n* = 8) and WT (*n* = 9) rats were transferred to the IntelliCage, which was programmed to study learning and memory-related behaviors over a period of 17 days, consisting of the following (Figure 1(b)):

1. Free exploration, in which animals were allowed to get familiar with the cage environment for 1 day: all doors were opened so animals have free access to the water bottle. The numbers of corner visits were counted to assess the exploratory activity and corner preference
2. Nosepoke learning, which lasted for 4 days: all doors were closed and rats must complete the nosepoke to open the door to access water. The numbers of corner visits and nosepokes were counted to assess the exploratory activity and corner preferences
3. Place learning, which lasted for a total of 8 days: the rat's least preferred corner of the nosepoke learning period was designated as “correct,” whilst the remaining corners were designated as “error.” All rats were able to visit all the corners, but only when the corner was “correct,” the door could be opened and drinking allowed. The place learning ability was measured by calculating the number of correct corner visits
4. Replace learning, which lasted for 4 days: the opposite corner of the “correct” corner in the position learning was designated as the new “correct” corner and the remaining corners were designated as “error.” Rats were allowed to visit all corners freely. Replace learning ability was measured by calculating the number of correct corner visits

#### 2.10.2. Anxiety-Related Behavior Test

Another cohort of ten-week-old female GPER^−/−^ (*n* = 6) and WT (*n* = 9) rats were transferred to the IntelliCage, which was programmed for evaluation of anxiety-like behaviors as follows:

1. Training period: in the first two days, rats were forbidden to drink water for 20 h each day (01:00-21:00) and then allowed to drink water for 4 h (21:00-01:00) but only in a specific corner
2. Testing period: in this stage, rats were forbidden to drink water for 20 h each day (01:00-21:00) and then allowed to drink for 4 h (21:00-01:00) in a specific corner. Each animal received a punitive air puff the first time it accessed water each day. This was repeated for 3 days. The average drinking latency (latency between the second drink and the first drink) and the average number of corner visits within the 4 h drinking period over the 3 days were calculated to measure the anxiety level of the rats

### 2.11. Statistics

Statistics analysis was performed using GraphPad PRISM 5. Numerical data are presented as mean ± SEM. Unpaired *t*-test was used to compare between two genotypes. To compare more than 2 groups, one-way or two-way ANOVA with Bonferroni or Tukey's post hoc test was performed. A *P* value less than 0.05 was considered as statistically significant.

## 3. Results

### 3.1. GPER Is Widely Distributed along the HPA Axis and in Brain Structures Involved in the Regulation of Anxiety

**Figure 2:**
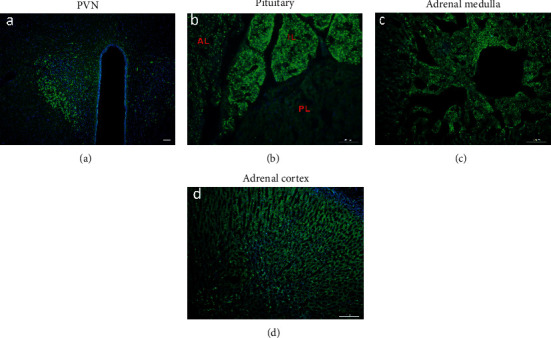
GPER immunoreactivity along the HPA axis in the rat. (a) GPER immunofluorescence (green) in the PVN. (b) GPER immunofluorescence in pituitary (AL = anterior lobe; IL = intermediate lobe; PL = posterior lobe). Note that GPER is enriched in the intermediate lobe (IL) of pituitary. (c) GPER immunofluorescence in the adrenal medulla. (d) GPER immunofluorescence in the adrenal cortex. Horizontal bars = 50 *μ*m.

**Figure 3:**
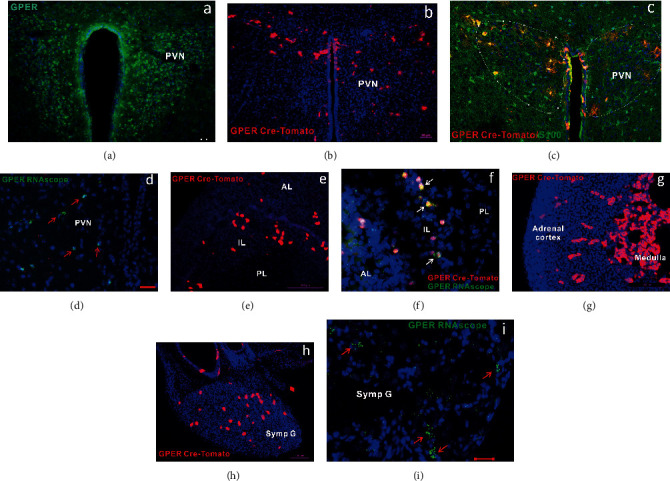
Distribution of GPER along the HPA axis in the mouse. (a) GPER immunofluorescence (green) in the PVN; (b) presence of Tomato^+^ cells (red) in the PVN of GPER reporter (GPER Cre/tdTomato) mice; (c) immunohistochemistry shows Tomato^+^ (red) cells express astrocyte marker S100 (green) in the PVN of GPER reporter mice. Note that only a fraction of S100^+^ cells are Tomato^+^. (d) GPER RNAscope in situ hybridization signal (green) in mouse PVN. (e) Presence of Tomato^+^ (red) cells in the intermediate lobe (IL) and anterior lobe (AL) but not in the posterior lobe (PL) of the pituitary. (f) GPER RNAscope signal (green) in the pituitary of GPER reporter mice. Note the presence of clustered RNAscope signal (green) in Tomato^+^ (red) cells in the intermediate lobe and weak sporadic RNAscope signal in the anterior lobe, but the absence of RNAscope signal in the posterior lobe. (g) Presence of Tomato^+^ cells in the adrenal gland. Note that most cells in the adrenal medulla are Tomato^+^ (red). Some cells in the adrenal cortex, particularly in the zona fasciculata, are Tomato^+^. (h) Presence of Tomato^+^ cells in the superior cervical sympathetic ganglion of GPER reporter mice. (i) GPER RNAscope signal in the superior cervical sympathetic ganglion of WT mice. Horizontal bars = 100 *μ*m.

The distribution of GPER along the HPA axis and in the prefrontal cortex, the hippocampal formation, and the amygdala was addressed by three complementary approaches: immunohistochemistry, GPER reporter mice, and RNAscope. Immunohistochemistry revealed widespread distribution of GPER immunofluorescence in these areas in rats and mice. Within the rat HPA axis, strong GPER immunofluorescence was detected in the paraventricular nucleus (PVN) of the hypothalamus, the intermediate lobe of the pituitary, and the adrenal medulla, whereas moderate GPER immunofluorescence was seen in the anterior lobe of the pituitary and the adrenal cortex (Figure 2). A similar pattern of GPER immunofluorescence was detected in the PVN (Figure 3(a)), the pituitary, and the adrenal medulla (data not shown) in the mice. The distribution of GPER immunofluorescence was consistent with the distribution of GPER/tdTomato cells in the GPER reporter mice. Thus, Tomato^+^ cells were clustered within the PVN (Figure 3(b)), the intermediate lobe of the pituitary (Figure 3(e)), and the adrenal medulla (Figure 3(g)), whereas sporadic Tomato^+^ cells were seen within the anterior lobe of the pituitary (Figure 3(e)) and adrenal cortex, especially in the zona fasciculata (Figure 3(g)). Tomato^+^ cells within the PVN were immunoreactive to S100 (Figure 3(c)), indicating that they were astrocytes rather than neurons. By RNAscope, GPER transcripts were clearly detected within the PVN (Figure 3(d)). Within the pituitary taken from GPER reporter mice, strong GPER RNAscope signal was detected in Tomato^+^ cells in the intermediate lobe, with weak GPER RNAscope signal being clearly visible in the anterior but not in the posterior lobe of the pituitary (Figure 3(f)). Additionally, in the GPER reporter mice, Tomato^+^ neurons were seen in the superior cervical sympathetic ganglion and this is consistent with positive GPER RNAscope signal being present in this ganglion (Figures 3(h) and 3(i)). These results indicate that estrogens may act via GPER at multiple levels to modulate the physiological and neuroendocrine responses to stress.

**Figure 4:**
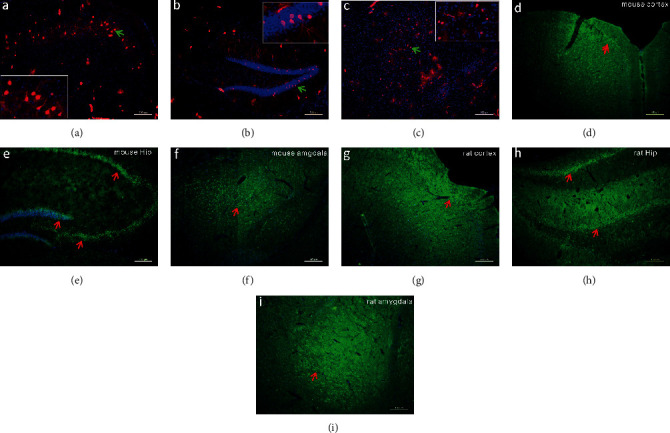
Distribution of GPER in the prefrontal cortex, hippocampus, and basal lateral amygdala. (a–c) Distribution of Tomato fluorescence (red) in the prefrontal cortex (PFC), the hippocampus (Hip), and the basolateral amygdala (BLA) in the GPER reporter (GPER Cre/tdTomato) mice. Green arrows indicate regions shown in higher magnification in the insets. (d–f) Distribution of GPER immunofluorescence (green) in mouse PFC, Hip, and BLA. Arrows point to positive staining. (g–i) Distribution of GPER immunofluorescence (green) in rat PFC, Hip, and BLA. Arrows point to positive staining. Horizontal bars = 100 *μ*m.

**Figure 5:**
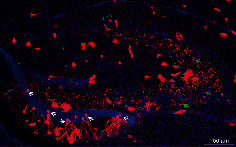
Distribution of the reporter gene (Tomato) in the hippocampal formation of the GPER reporter mice. Tomato^+^ neuronal cell bodies (white arrows) are localized in the dentate gyrus (DG) but not in the hilum, CA3, CA2, or CA1 regions, where Tomato^+^ fibers and terminal boutons (green arrows) are numerous.

**Figure 6:**
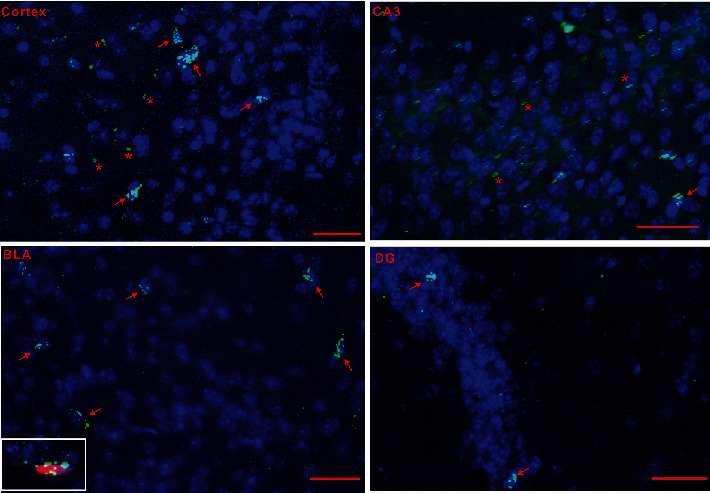
Distribution of GPER RNAscope in situ hybridization signal (green) in the prefrontal cortex, hippocampus (CA3 and DG regions), and basal lateral amygdala (BLA). Red arrows point to clustered signal indicative of expression in cell bodies. Red stars indicate weak sporadic RNAscope signal presumably in fiber terminals. The inset shows a representative Tomato^+^ neuronal body in the BLA of GPER reporter mice with strong clustered RNAscope signal. Horizontal bars = 50 *μ*m.

The prefrontal cortex, the hippocampal formation, and the amygdala are critically involved in the cognitive and behavioral responses to stress. We found that these structures were enriched with GPER immunofluorescence, both in mice and in rats (Figures 4(d)–4(i)). Consistently, Tomato^+^ cells were present in these structures in the GPER reporter mice (Figures 4(a)–4(c)). Interestingly, within the hippocampal formation, whilst GPER immunofluorescence seemed to be ubiquitous in the dentate gyrus (DG), CA1, CA2, and CA3 regions (Figures 4(e) and 4(h)), Tomato^+^ neuronal bodies were only seen in DG but not in CA1, CA2, CA3, and the hilum regions, where instead dense Tomato^+^ terminal fibers and synaptic boutons were clearly visible (Figure 5). GPER RNAscope signal was also detected in the prefrontal cortex (Figure 6(a)), basolateral amygdala (BLA, Figure 6(b)), CA3 (Figure 6(c)), and DG (Figure 6(d)). It was interesting to note that clustered RNAscope signal (likely present in neuronal bodies) was seen in the prefrontal cortex, BLA, and DG, whereas sporadic RNAscope signal (presumably present in terminal fibers) was detected in CA3, which was consistent with the distribution of Tomato^+^ cell bodies and fibers within these regions. These results revealed widespread but unique pattern of distribution of GPER within brain structures implicated in the regulation of anxiety.

### 3.2. GPER-Deficient Rats Had Lower Basal Serum Corticosterone Levels

**Figure 7:**
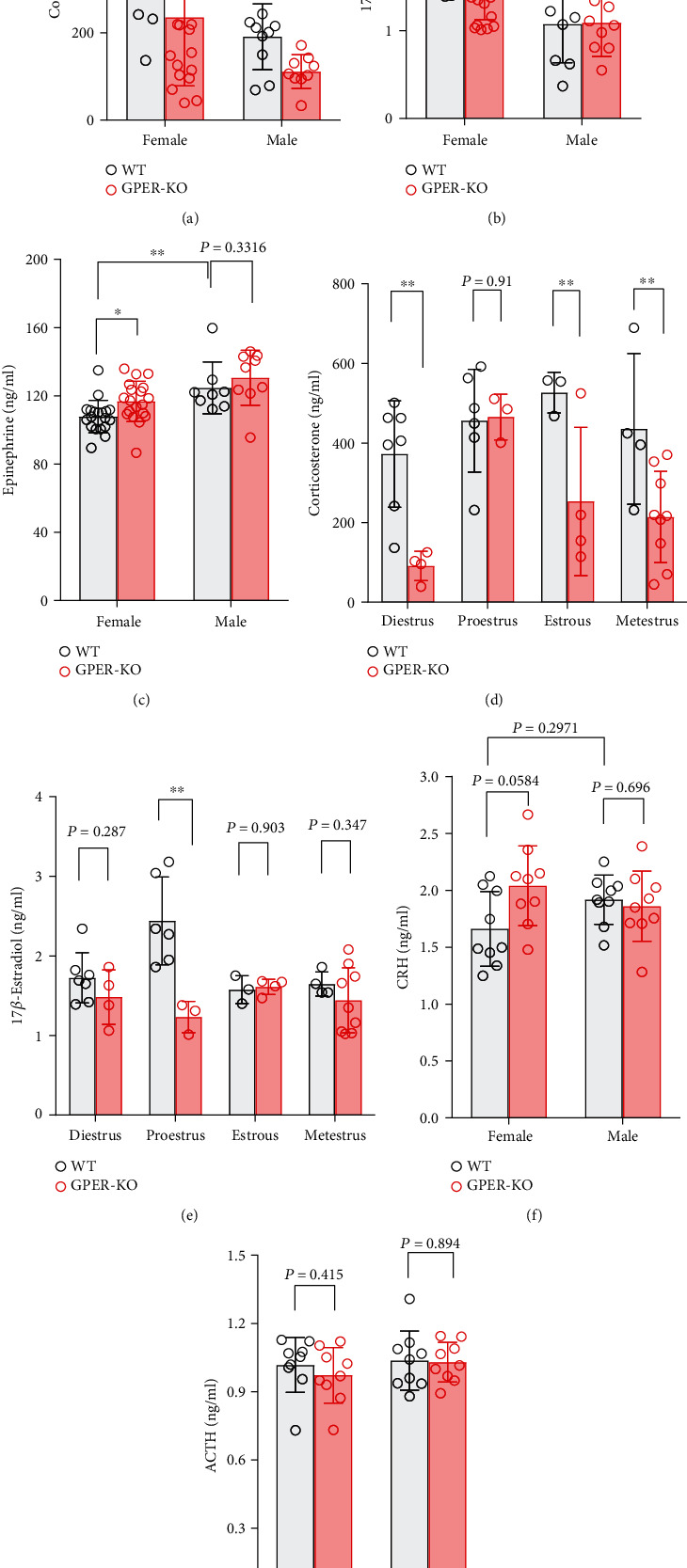
GPER-deficient rats had lower basal serum corticosterone level. (a) Basal serum corticosterone level (detected by HPLC-MS) in WT and GPER^−/−^ rats of either sex. Note that serum corticosterone of GPER^−/−^ female rats (*n* = 20) is significantly lower than that of WT female rats (*n* = 20). GPER^−/−^ male rats (*n* = 10) also seemed to have lower serum corticosterone level than WT males (*n* = 10), but the difference did not reach statistical significance. (b) Basal serum17*β*-estradiol level (detected by HPLC-MS) in WT and GPER^−/−^ rats of either sex. Note that serum 17*β*-estradiol of GPER^−/−^ female rats (*n* = 20) is significantly lower than that of WT female rats (*n* = 20). (c) Basal serum adrenaline level (detected by HPLC-MS) in WT and GPER^−/−^ rats of either sex. Note that serum adrenaline of GPER^−/−^ female rats (*n* = 20) is significantly higher than that of WT female rats (*n* = 20). (d) Basal serum corticosterone level in WT and GPER^−/−^ rats of different menstrual cycle. (e) Basal serum 17*β*-estradiol level in WT and GPER^−/−^ rats of different menstrual cycle. (f) Basal plasma CRH level (detected by ELISA) in WT and GPER^−/−^ rats of either sex. Note that plasma CRH of GPER^−/−^ female rats (*n* = 10) is significantly higher than that of WT female rats (*n* = 10). (g) Basal plasma ACTH level (detected by ELISA) in WT and GPER^−/−^ rats of either sex. ∗*P* < 0.05, ∗∗*P* < 0.01, ∗∗∗*P* < 0.001, and ∗∗∗∗*P* < 0.0001, two-way ANOVA with Tukey post hoc test.

Given the widespread distribution of GPER within the HPA axis and the sympathetic nervous system (adrenal medulla and sympathetic ganglia), we next investigated whether GPER deficiency may impact serum or plasma levels of stress hormones. Consistent with previous reports [34], WT female rats had significantly higher basal serum corticosterone but lower basal adrenaline levels than WT male rats (Figures 7(a) and 7(c)). Strikingly, the basal serum corticosterone level of GPER-deficient (GPER^−/−^) female rats was markedly lower than that of WT female rats (Figure 7(a)), whereas serum adrenaline level was slightly but significantly increased in GPER^−/−^ female rats as compared to the WT female rats (Figure 7(c)). It was interesting to note that GPER^−/−^ male rats also had a lower basal corticosterone level than WT male rats (Figure 7(a)), but serum adrenaline levels were not significantly different between GPER^−/−^ and WT male rats (Figure 7(c)). Plasma level of CRH was slightly increased in GPER^−/−^ female rats as compared with the WT female rats (Figure 7(f)), which was likely explained by decreased negative feedback (i.e., due to lower serum corticosterone level). However, GPER deficiency did not significantly affect plasma ACTH levels either in female or in male rats (Figure 7(g)). These results imply that peripheral GPER (i.e., GPER in the adrenal cortex) might be responsible for the higher basal corticosterone level in female than in male rats.

GPER^−/−^ female rats had significantly lower serum E2 in the proestrus phase than the WT female rats, but no difference in serum E2 level was found between GPER^−/−^ and WT male rats (Figures 7(b) and 7(e)). We also analyzed the impact of GPER deficiency on serum corticosterone levels at different phases of the menstrual cycle, with lower levels found in diestrus, estrous, and metestrus phases but not in proestrus phase in GPER^−/−^ rats compared with the WT rats (Figure 7(d)).

**Figure 8:**
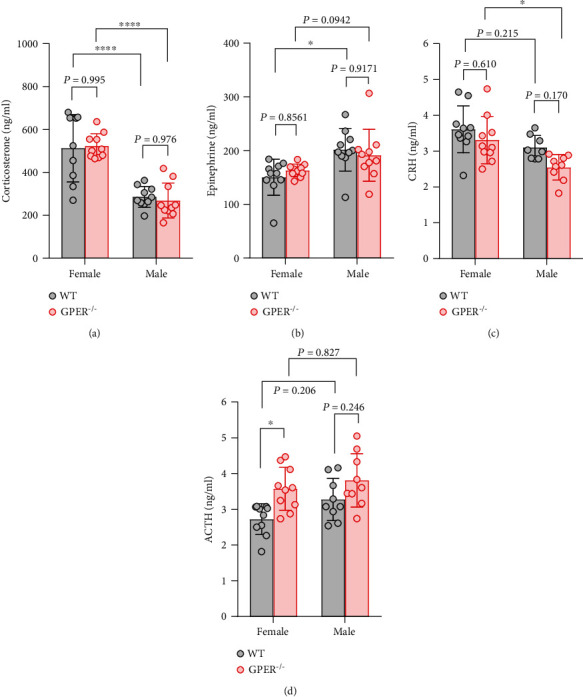
Serum levels of stress hormones following acute restraint stress in wild-type and GPER-deficient rats. (a) Serum corticosterone level following 30 min acute restraint stress in WT and GPER^−/−^ rats of either sex (*n* = 10 for each group). (b) Serum adrenaline level in WT and GPER^−/−^ rats of either sex following acute restraint stress. (c) Plasma CRH level in WT and GPER^−/−^ rats of either sex following acute restraint stress. Note that GPER^−/−^ rats (*n* = 10) of either sex seemed to have lower plasma CRH level than WT (*n* = 10) following acute restraint stress, but the difference did not reach statistical significance. (d) Plasma ACTH level in WT and GPER^−/−^ rats of either sex following acute restraint stress. Note that GPER^−/−^ rats had higher plasma ACTH level than the WT rats, particularly in the female. ∗*P* < 0.05 and ∗∗∗∗*P* < 0.0001, two-way ANOVA with Tukey post hoc test.

Given the markedly lower basal corticosterone level in GPER^−/−^ than WT rats, we wondered how GPER deficiency would affect HPA axis responses to stress. Therefore, in another cohort of GPER^−/−^ and WT rats, we measured the serum or plasma levels of stress hormones following 30 min of restraint stress. Surprisingly, neither serum corticosterone nor adrenaline levels were significantly different between GPER^−/−^ and WT female or between GPER^−/−^ and WT male rats following acute restraint stress (Figures 8(a) and 8(b)). However, plasma ACTH level was significantly higher in GPER^−/−^ female than in WT female rats following the acute stress, despite no significant difference in plasma CRH levels between these two groups (Figures 8(c) and 8(d)). The results imply that GPER at the level of the pituitary may negatively regulate stress responses of the HPA axis, such that removal of this negative modulation (in GPER^−/−^ rats) may enhance ACTH and corticosterone release during acute stress.

### 3.3. GPER-Deficient Rats Display Increased Anxiety-Like Behaviors

**Figure 9:**
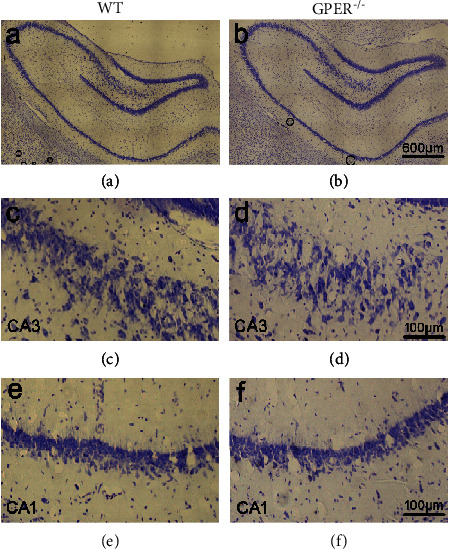
GPER deficiency in the rat did not significantly affect hippocampal morphology. (a, b) Nissl-stained hippocampal formation of WT and GPER^−/−^ rats. (c, d) Nissl-stained CA3 region of WT and GPER^−/−^ rats. (e, f) Nissl-stained CA1 region of WT and GPER^−/−^ rats.

Seeing that GPER is widely distributed in the corticolimbic circuit comprising the prefrontal cortex, the hippocampal formation, and the amygdala, we next investigated whether GPER deficiency may impact animal behaviors related to mood, learning, and memory. It is worthy of mentioning here that Nissl staining indicated that hippocampal morphology of GPER^−/−^ rats was intact compared with the WT rats (Figure 9).

**Figure 10:**
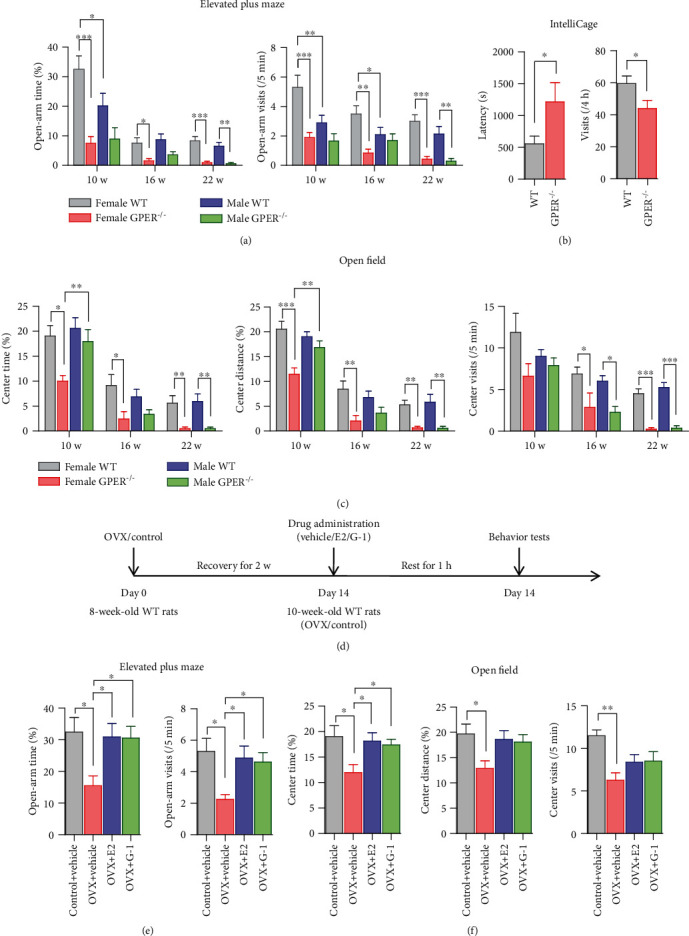
GPER-deficient rats exhibit anxiety-like behavior. (a) Comparison of the open-arm time and open-arm visits in the elevated plus maze (EPM) between WT and GPER^−/−^ rats of three age groups (10 weeks old, *n* = 10; 16 weeks old, *n* = 10; and 22 weeks old, *n* = 7). (b) Comparison of the latency to drink and corner visits after punitive air puff in the IntelliCage between WT (*n* = 9) and GPER^−/−^ (*n* = 6) female rats. (c) Comparison of the center time, center distance, and center visits in the open field test between WT and GPER^−/−^ rats of three age groups (10 w, *n* = 8; 16 w, *n* = 8; and 22 w, *n* = 8). (d) Protocol of the OVX test to further explore the role of GPER in modulation of anxiety. (e) Comparison of the open-arm time and open-arm visits in the EPM test among control (*n* = 10), ovariectomized (OVX, *n* = 10), and OVX rats treated with E2 (*n* = 10) or G-1 (*n* = 10). Note that OVX rats showed anxiety-like behaviors, which were reversed by E2 and G-1. (f) Comparison of the center time, center distance, and center visits in the open field test among control (*n* = 8), OVX (*n* = 8), and OVX rats treated with E2 (*n* = 8) or G-1 (*n* = 8). Note that E2 and G-1 can reverse OVX-induced anxiety-like behaviors. ∗*P* < 0.05, ∗∗*P* < 0.01, and ∗∗∗*P* < 0.001, two-way ANOVA with Bonferroni posttests for elevated plus maze and open field, unpaired *t*-test for IntelliCage.

Anxiety-like behaviors were investigated by elevated plus maze (EPM), open field, and IntelliCage tests. In the EPM test, GPER^−/−^ female rats of three age groups (10, 16, and 22 weeks) all showed remarkable decreases in open-arm time (percentage of time spent in the open arm) and number of open-arm visits compared with their WT counterparts (Figure 10(a)). In the 10- and 16-week-old male rats, open-arm time or number of open-arm visits was not significantly different between GPER^−/−^ and WT groups. However, in the 22-week-old male rats, there were significant decreases in open-arm time and number of open-arm visits in GPER^−/−^ than the WT controls (Figure 10(a)). Similar results were obtained in the open field test. Thus, center time, center distance, and center visits were all decreased in female GPER^−/−^ compared with their WT counterparts in all three age groups (Figure 10(c)). For the male rats, only the 22-week-old GPER^−/−^ group showed significant decreases in center time and center distance compared with the WT control group (Figure 10(c)). The IntelliCage test, with the advantage of enabling evaluation of animal's anxiety level in a social environment, was carried out in 10-week-old female WT and GPER^−/−^ rats. The animals were trained to drink water at a specific corner, and on the testing days, they received a punitive air puff when they first drank water after 20 h of water deprivation. The latency taken for the animals to access water again (drinking latency) and frequency of access (visits) during a 4 h period were registered. GPER^−/−^ rats were found to have significantly longer drinking latency and fewer visits compared with their WT counterparts (Figure 10(b)). These results demonstrated that GPER deficiency, particularly in the females, may lead to behaviors indicative of increased anxiety level in the rats.

To further explore the role of GPER in the modulation of anxiety, we conducted EPM and open field tests to observe the effects of ovariectomy (OVX) and systemically administered E2 (endogenous GPER agonist, 10 *μ*g/kg, s.c.) or G-1 (synthetic GPER agonist, 10 *μ*g/kg, s.c.) on anxiety-like behaviors in 10-week-old female rats. As shown in Figures 10(e) and 10(f), OVX resulted in behaviors indicative of increased anxiety (less open-arm time and fewer open-arm visits in EPM test; less center time and center distance and fewer center visits in open field test), which were reversed by E2 and G-1 (Figures 10(e) and 10(f)).

**Figure 11:**
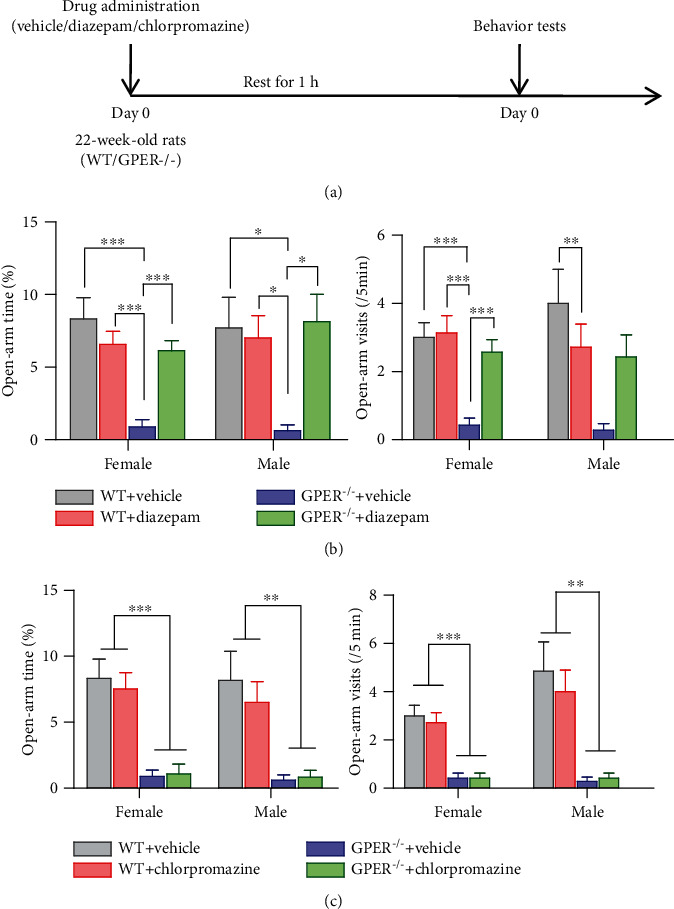
Diazepam reverses anxiety-like behavior in GPER-deficient rats. (a) Protocol of the drug administration test to explore the possible neurotransmitter which can be the mechanistic basis of the anxiety-like behavior of GPER^−/−^ rats. (b) Comparison of the open-arm time and open-arm visits in the elevated plus maze test in WT and GPER^−/−^ rats treated with diazepam or vehicle (*n* = 7 for each group). Note that diazepam can reduce the anxiety-like behavior of GPER^−/−^ rats. (c) Comparison of the open-arm time and open-arm visits in the elevated plus maze test in WT and GPER^−/−^ rats treated with chlorpromazine or vehicle (*n* = 7 for each group). Note that chlorpromazine did not seem to affect the anxiety-like behavior of GPER^−/−^ rats. ∗*P* < 0.05, ∗∗*P* < 0.01, and ∗∗∗*P* < 0.001, two-way ANOVA with Bonferroni post hoc tests.

Imbalanced excitatory and inhibitory neurotransmissions are regarded as an important mechanism underlying anxiety disorders [35, 36], which is the mechanistic basis of mainstream medications such as diazepam (GABA_A_ agonist) and chlorpromazine (dopamine receptor antagonist). We wondered whether diazepam or chlorpromazine may affect the anxiety-like behavior of GPER^−/−^ rats. To answer such a question, WT and GPER^−/−^ rats of either sex were injected with diazepam (1 mg/kg, ip) or chlorpromazine (1 mg/kg, ip) 1 h before the EPM test. Diazepam did not significantly alter open-arm time or open-arm visits in the WT rats. In the GPER^−/−^ rats, however, diazepam caused significant increases in open-arm time and open-arm visits (Figure 11(b)). In contrast, chlorpromazine had no significant effect on open-arm time or open-arm visits either in WT or GPER^−/−^ rats (Figure 11(c)).

### 3.4. GPER Deficiency Accentuates Anxiety-Like Behavior and Alters Neuroendocrine Profile following Single-Prolonged Stress

**Figure 12:**
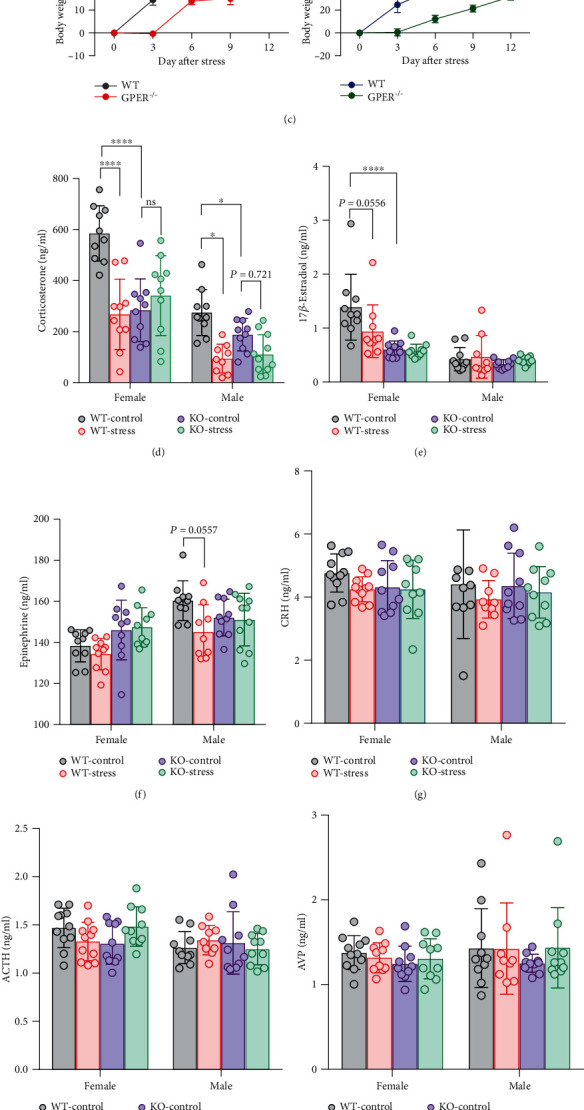
GPER deficiency accentuates anxiety-like behaviors and alters neuroendocrine profile following single-prolonged stress. (a) Timeline of the SPS model and the following tests to explore whether GPER deficiency in the rat may alter the anxiety-like behavior and the neuroendocrine profile following stress. (b) Comparison of open-arm time and open-arm visits in the elevated plus maze (EPM) between unstressed or stressed (with single-prolonged stress (SPS)) WT and GPER^−/−^ rats of either sex. *n* = 10 for each unstressed group, *n* = 7 for each stressed group. Note that stressed GPER^−/−^ rats of either sex were least likely to visit or to stay in the open arm, indicative of higher anxiety levels, compared with other groups. (c) Comparison of the body weight gain between WT (*n* = 7 each gender) and GPER^−/−^ (*n* = 7 each gender) rats following SPS. (d–j) Comparison of serum or plasma levels of corticosterone, 17*β*-estradiol, adrenaline, CRH, ACTH, vasopressin (AVP), and *β*-endorphin between unstressed or stressed WT and GPER^−/−^ rats (*n* = 10 for each group). Note that SPS caused a significant decrease in serum corticosterone levels and slightly lower serum adrenaline levels in WT but not GPER^−/−^ rats. ∗*P* < 0.05, ∗∗*P* < 0.01, and ∗∗∗*P* < 0.001, two-way ANOVA with Bonferroni or Tukey post hoc tests.

Women are more likely than men to develop posttraumatic stress disorders (PTSD) following life-threatening tragic events [37], and a dysfunctional HPA axis has been implicated in the pathogenesis of PTSD [38, 39]. We wondered whether GPER deficiency in the rat may alter the anxiety-like behavior and the neuroendocrine profile following an episode of intense stress. To answer such a question, 8-week-old GPER^−/−^ and WT rats of either sex were subjected to single-prolonged stress (SPS). Following 2 weeks of quiescence period, they were tested for anxiety-like behaviors on EPM and serum or plasma levels of stress hormones. In female WT rats, the stressed group had significantly less open-arm time (reduced by 73%) and fewer open-arm visits (reduced by 57%) than the unstressed (WT control) group (Figure 12(b)). In female GPER^−/−^ rats, the stressed group barely visited or stayed in the open arm (open-arm time reduced by 94% and open-arm visits reduced by 92%) (Figure 12(b)). Similar results were found in male rats with the stressed GPER^−/−^ group being least likely to visit or stay in the open arm compared with other groups (Figure 12(b)). These results indicate that GPER deficiency may accentuate anxiety-like behaviors following SPS.

Interestingly, we noted that GPER^−/−^ rats of either sex showed significantly less body weight gain than their WT counterparts following SPS and this was primarily evident in the first 3 days after stress, when GPER^−/−^ rats showed zero weight gains (Figure 12(c)).

In line with the literature [40], we found that 2 weeks after SPS, WT rats of either sex had significantly lower serum corticosterone level compared with the unstressed controls (Figure 12(d)). In contrast, the serum corticosterone level of stressed GPER^−/−^ female rats was comparable with that of the unstressed GPER^−/−^ female group (Figure 12(d)). The serum corticosterone level of the stressed GPER^−/−^ male group seemed to be lower than that of the unstressed control group, but the difference did not reach statistical significance (Figure 12(d)). In addition, stressed WT female rats had lower serum 17*β*-estradiol than unstressed WT females (Figure 12(e)), whilst stressed WT male rats had lower serum adrenaline than unstressed WT males (Figure 12(f)). Such differences were not seen in GPER^−/−^ rats (Figures 12(e) and 12(f)). We did not find significant effects of SPS or GPER deficiency on plasma levels of CRH, ACTH, vasopressin (AVP), or *β*-endorphin (Figures 12(g)–12(j)). However, it was noted that SPS led to slightly lower plasma CRH, ACTH, and AVP levels in WT but not in GPER^−/−^ rats.

### 3.5. GPER-Deficient Rats Display Impaired Learning and Memory

Morris water maze (MWM) and IntelliCage tests were conducted to evaluate learning and memory-related behavior. In MWM test, GPER^−/−^ rats, whether female or male, showed significantly longer latency to find the platform compared with their WT counterparts during the 5-day positioning navigation tests (Figure 1(a)). On the 6^th^ day (spatial exploration test), GPER^−/−^ female and male rats showed slightly lower percentage of time in the target quarter than their WT counterparts, but the differences did not reach statistical significance (Figure 1(a)).

In the IntelliCage test (Figure 1(b)), GPER^−/−^ and WT rats showed similar corner visits during the free exploration period (Figure 1(c)). During the 4-day nosepoke learning period, GPER^−/−^ rats had fewer corner visits than the WT rats (Figure 1(d)), indicative of decreased simple skill learning ability in GPER^−/−^ rats. GPER^−/−^ rats had fewer numbers of licks and nosepokes than the WT rats on the first day of nosepoke learning. Lastly, during the place and replace learning period, GPER^−/−^ rats showed higher error rates than the WT rats, indicative of decreased spatial learning and memory (Figures 1(e) and 1(f)). These results demonstrated that GPER-deficient rats had impaired learning and memory.

## 4. Discussion

Organizational and activational effects of estrogens are presumably responsible for the gender difference in the stress responses and the higher prevalence of stress-related disorders in females [2, 3, 7]. Estrogens may not only act via the nuclear receptors ER*α* and ER*β* to mediate classical slow genomic effects but also bind to GPER to mediate rapid nongenomic effects. The current investigation has systematically analyzed the distribution and function of GPER in the corticolimbic circuit and the HPA axis. Our findings support GPER as a major player in mediating the estrogenic influences on the HPA axis and anxiety.

We took three complementary approaches to analyze GPER expression in the HPA axis and the corticolimbic circuit at transcription and protein levels. GPER immunofluorescence could be detected at every level of the HPA axis, with high expressions seen in the PVN and the intermediate lobe of pituitary and moderate expressions seen in the anterior pituitary and adrenal cortex in rats and mice, which was consistent with some previous reports [26, 41]. Importantly, GPER reporter (GPER Cre/tdTomato) mice and GPER RNAscope *in situ* hybridization revealed similar pattern of GPER expression at the transcription level. Within the corticolimbic circuit, moderate GPER immunofluorescence was ubiquitously present in the prefrontal cortex, the hippocampal formation, and the basolateral amygdala, which was in agreement with previous findings [26, 41]. GPER reporter mice and GPER RNAscope confirmed GPER transcription in these structures. The distribution pattern of Tomato and RNAscope signal in the hippocampal formation seemed to suggest that GPER-expressing granular neurons in DG may project extensively to CA3, CA2, CA1, and the hilum regions, where GPER may be expressed presynaptically. These results confirm that GPER is well positioned to mediate rapid estrogenic effects on the corticolimbic circuit [28] and the HPA axis.

It is well documented that females generally have higher basal and stress-induced glucocorticoid levels but lower basal and stress-induced adrenaline level than males [2, 20]. We argue that GPER may be primarily responsible for such gender differences, since the loss of GPER caused a dramatic decrease in basal serum corticosterone and a significant increase of basal serum adrenaline in female rats. We reason that GPER within the adrenal cortex facilitates basal corticosterone secretion since basal plasma concentration of ACTH was not significantly different between GPER^−/−^ and WT female rats. It may also be likely that GPER in the adrenal cortex inhibits adrenaline secretion, thereby contributing to the lower basal serum adrenaline level in females than in males. The profile of stress hormones following acute restraint stress was suggestive of an inhibitory role of GPER at the level of the pituitary on the HPA reactivity to stress. Thus, despite the significantly lower basal corticosterone level, GPER^−/−^ rats had similar serum level of corticosterone and concomitantly greater ACTH (but not CRH) response compared with the WT rats. Interestingly, physiologically relevant doses of E2 reportedly inhibit ACTH release but significantly increase adrenal sensitivity in OVX female rats [42]. It seems possible that those effects may be mediated by GPER.

We conducted the elevated plus maze (EPM), open field, and IntelliCage tests to explore the effects of GPER deficiency on behaviors related to anxiety. Seeing that besides gender, age is also an important factor in anxiety [43–45], we studied male and female rats of three age groups (10, 16, and 22 weeks of age, which correspond to adolescent, adult, and middle age of humans). GPER^−/−^ rats of all three age groups displayed behaviors indicative of increased anxiety particularly in the females. The greater effects seen in the female were suggestive of the involvement of circulating estrogens in mood regulation via GPER, which was further supported by the findings that OVX rats showed behaviors indicative of increased anxiety, which could be reversed by subcutaneous administration of E2 or G-1. An earlier study also showed that GPER agonists ameliorated anxiety-like behaviors in rats [46]. These results indicate that GPER generally mediates a positive anxiolytic effect. However, there have also been reports that systemic G-1 led to decreased anxiety-like behaviors in gonadectomized male but not female mice [29] or led to increased anxiety level in OVX mice [30]. In addition, GPER-deficient male but not female mice reportedly displayed reduced anxiety-like behaviors [31]. The inconsistencies are not surprising, given the widespread distribution of GPER in the corticolimbic regions and the HPA axis. It may well be possible that GPER at different sites may modulate anxiety differently.

Given the distinct distribution of GPER in the hippocampal formation, we also investigated whether GPER deficiency may affect learning and memory. Morris water maze and IntelliCage tests showed impaired learning and spatial memory in the GPER^−/−^ female rats. There have been reports that the activation of GPER may improve the performance of rats in T-maze task and the inhibition of GPER has the opposite effect [47, 48]. Therefore, GPER appears to play a favorable role promoting learning and memory.

In summary, the present study has revealed widespread expression of GPER in the corticolimbic circuit, the HPA axis, and the sympathetic ganglia in rats and mice. GPER appears to play a major role in mediating gender differences in the HPA axis and in regulation of the cognitive, autonomic, and neuroendocrine responses to stress. Since GPER deficiency in the rat resulted in significant phenotypes including altered stress hormone profile in basal and stressed conditions, vulnerability to homeostatic disturbance such as reduced body weight gain and hypertension following stress [33], increased anxiety-like behaviors, and impaired learning and memory, more detailed analysis of GPER at different levels is warranted.

## Data Availability

All data supporting the results of this study are included in the article.
